# AI-Driven Approaches for the Detection, Classification, and Surveillance of Viral Pathogens: Current Advances, Challenges, and Future Directions

**DOI:** 10.3390/pathogens15070761

**Published:** 2026-07-20

**Authors:** Hathem Khelil, Rosanna Palumbo, Giovanni N. Roviello

**Affiliations:** 1 Laboratory of Informatics and Its Applications (LIAM), Mohamed Boudiaf University, M’sila 28000, Algeria; 2 Institute of Biostructures and Bioimaging (IBB), National Research Council (CNR), Via T. De Amicis, 80134 Naples, Italy

**Keywords:** AI-based viral detection, machine learning in virology, image-based diagnostics, outbreak surveillance, viral diseases

## Abstract

Artificial intelligence (AI) has rapidly emerged as a transformative tool in virology, offering new opportunities for the detection, classification, and surveillance of viral pathogens. Recent advances in machine learning, deep neural networks, and multimodal data analysis now enable the identification of viral signatures from genomic sequences, medical images, environmental samples, and social-media-derived epidemiological signals. This review provides a comprehensive overview of state-of-the-art AI methodologies applied to viral pathogen research, with a particular focus on image-based diagnostics, automated quality assessment of virology-related digital content, and predictive modelling for outbreak monitoring. We discuss how convolutional and transformer-based architectures are being used to classify infected tissues, detect viral particles, and support laboratory workflows. Furthermore, we highlight the emerging role of AI in evaluating the reliability of user-generated images and short videos related to infectious diseases, an area increasingly relevant in the age of misinformation. Challenges such as dataset bias, limited annotated virological images, ethical concerns, and the need for standardized quality-assessment pipelines are critically examined. Finally, we outline future research directions, including hybrid AI–biological models, AI-supported viral surveillance in healthcare environments, and the integration of explainable AI to enhance clinical trust.

## 1. Introduction

Viral pathogens continue to pose major challenges to public health, causing substantial morbidity, mortality, and economic disruption globally. Recent outbreaks of emerging and re-emerging viruses, including severe acute respiratory syndrome coronavirus 2 (SARS-CoV-2) [1], Ebola virus [2], Zika virus [3], influenza viruses [4], and other respiratory and vector-borne pathogens, have highlighted the urgent need for rapid diagnostic tools, more effective surveillance systems, and timely public health interventions. On the other hand, advances in techniques such as genomic sequencing [5], medical imaging [6], and digital epidemiology [7] have generated unprecedented volumes of clinical and biological data, creating new opportunities for computational approaches capable of extracting meaningful insights from the obtained complex datasets. In virology, these rapidly expanding resources are particularly useful as they encompass viral genome sequences, functional and structural protein data, electronic health records, medical imaging, and epidemiological surveillance data. The integration and analysis of these diverse data streams using artificial intelligence (AI) have facilitated viral pathogen characterization, biomarker discovery, outbreak monitoring, and the identification of novel therapeutic targets, thereby strengthening both research and public health preparedness.

Remarkably, artificial intelligence, encompassing machine learning (ML) and deep learning (DL) methodologies, has emerged as a transformative technology across biomedical and healthcare research [8]. AI-based systems can identify complex patterns within imaging, genomic, and epidemiological data that may not be readily detectable using traditional analytical approaches. Within the field of virological investigation, these methods have demonstrated considerable promise for different applications including pathogen detection, viral genome analysis, disease classification, outbreak forecasting, medical image interpretation, and public health surveillance [9,10,11]. The increasing availability of high-performance computing resources, together with the growing abundance of large-scale biological datasets, has markedly accelerated the adoption of AI-driven approaches in infectious disease research. These advances have enabled the integration and analysis of diverse clinical, imaging, genomic, and epidemiological data, providing new opportunities to improve disease diagnosis, pathogen surveillance, and therapeutic development [12].

Recent advances in artificial intelligence have also expanded beyond diagnostic and surveillance applications into the molecular investigation of viral pathogens. Deep-learning frameworks, protein language models, and structure-prediction systems have enabled accurate characterization of viral proteins and prediction of host–virus interactions [13,14,15,16]. These advances have also accelerated the identification of potential therapeutic targets and antiviral drug discovery [17,18].

At the same time, numerous challenges persist with respect to data quality, model interpretability, reproducibility, generalizability, and ethical deployment. Many AI models rely on large annotated datasets that may not adequately represent diverse populations, geographic regions, or emerging pathogens. Furthermore, specific concerns regarding explainability and transparency continue to influence the adoption of AI systems in public health and clinical settings. Consequently, addressing these limitations is essential to ensure that AI-driven technologies can be deployed responsibly and reliably.

This review provides an overview of contemporary AI applications in virology, focusing on viral pathogen detection, classification, molecular characterization, digital-content quality assessment, and outbreak surveillance. Current developments, methodological challenges, and future research directions are discussed, highlighting the expanding role of artificial intelligence in improving virological research, public health preparedness, and antiviral therapeutic development.

### Methodology

Relevant literature for the present work was identified through searches of multiple databases, including PubMed, Scopus, Web of Science, and Google Scholar, covering the period 1 January 2012 to 30 June 2026. The literature search included peer-reviewed research articles, review articles, and methodological studies published in English. Search terms were used individually or in combination and included: “viral pathogens”, “virus detection”, “virus classification”, “genomic surveillance”, “artificial intelligence”, “machine learning”, “deep learning”, “outbreak forecasting”, “epidemic prediction”, “protein structure prediction”, “host–virus interactions”, “antiviral drug discovery”, “medical imaging”, “misinformation detection”, “digital epidemiology”, and “multimodal learning”. The studies incorporated in this review were published between 2012 and 2026, with most appearing after 2020, highlighting the growing contemporary interest in this research area.

**Scheme 1:**
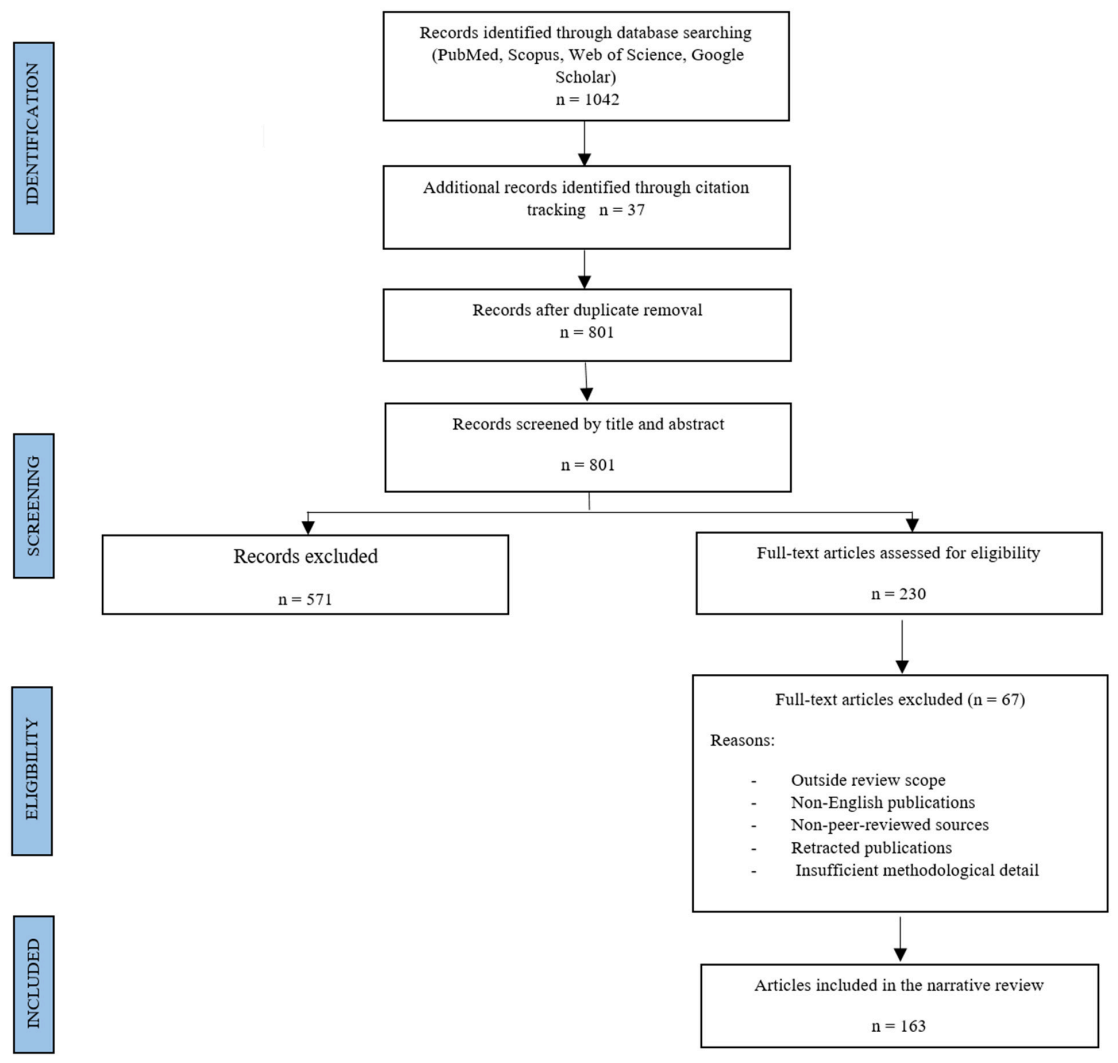
PRISMA 2020 flow diagram of the literature search and study selection for this review (edited by the authors using the Canva online platform, https://www.canva.com/, accessed on 11 July 2026).

In the literature search, priority was given to studies that addressed relevant virological or public health challenges, demonstrated well-defined methodologies, and provided meaningful advances in surveillance, diagnostic, predictive, or therapeutic applications, and these elements were incorporated into our inclusion criteria. Exclusion criteria included retracted papers, duplicates, non-English publications, non–peer-reviewed sources, studies lacking methodological detail, and works unrelated to computational virology. Additional references were identified through citation tracking of relevant publications, resulting in a final set of 163 included references (Scheme 1).

## 2. AI for Viral Detection and Classification from Genomic and Imaging Data

**Figure 1:**
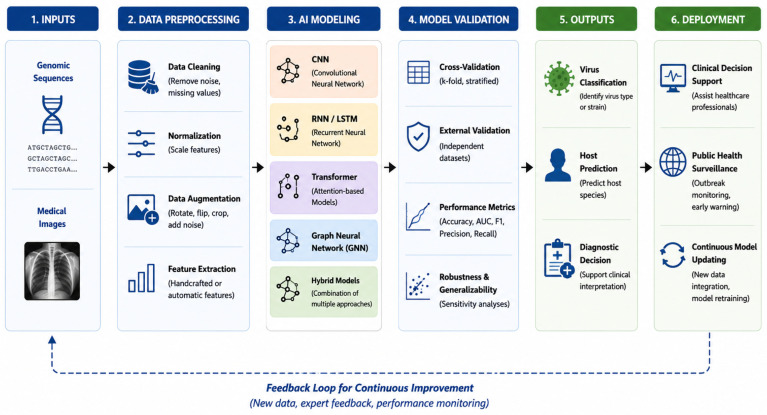
Overview of AI-driven viral pathogen detection and classification workflows having as outputs the virus classification, the host prediction and the diagnostic decision (image created by the authors using the Canva online platform, https://www.canva.com/, accessed on 11 July 2026).

This section examines established artificial intelligence approaches for the detection and classification of viral pathogens using genomic sequences and biomedical imaging data. It covers supervised and deep learning models for viral genome annotation, host–virus interaction prediction, and taxonomic classification, as well as convolutional and transformer-based architectures applied to diagnostic techniques such as microscopy, histopathology, and radiological imaging. Particular attention is devoted to methods supported by benchmark datasets, standardized evaluation metrics, and experimentally validated performance, while also highlighting key methodological limitations and emerging research gaps (Figure 1).

### 2.1. Genomic-Based Viral Detection and Classification

Artificial intelligence techniques have become central to viral genomics, particularly for identifying and classifying pathogenic viral sequences from high-throughput sequencing data. Early machine learning approaches relied on handcrafted features such as k-mer frequencies combined with classifiers including support vector machines and random forests. While effective in controlled datasets, these approaches often lacked generalization across diverse viral taxa [19,20].

Latest innovations have shifted toward deep learning architectures capable of automatically extracting hierarchical representations from raw nucleotide sequences. Convolutional neural networks and recurrent neural networks have demonstrated improved performance in viral classification and detection tasks, particularly for identifying novel pathogens and resolving taxonomic ambiguities [21,22]. In recent years, transformer-based models such as DNABERT [23] and protein-focused transformers [24] have been introduced, enabling the modeling of long-range dependencies in genomic sequences and improving classification accuracy across complex viral families.

In addition to classification, AI approaches have been applied to host prediction and virus–host interaction analysis. These methods are essential for understanding viral transmission pathways as well as zoonotic risks. Recent research integrating sequence-based features with protein interaction networks has demonstrated promising performance, although their effectiveness is still limited by the availability of experimentally validated datasets [25,26].

In this context, applications have extended to Hepatitis C virus (HCV), where machine learning has been used for treatment outcome prediction [27] and AI-assisted diagnostic biomarker identification [28], Human Immunodeficiency Virus (HIV), where AI supports drug resistance prediction [29] and transmission modeling [30]; Ebola virus, where AI-assisted analysis of portable genome sequencing data supports real-time genomic surveillance [31], and oncogenic Human Papillomavirus (HPV) and Herpes Simplex Virus (HSV), where deep learning aids histopathology and lesion classification [32]. AI has also been applied to HSV replication imaging using CRISPR-based techniques [33]. The above examples highlight the versatility of AI across diverse viral domains.

Despite these advances, genomic AI models face several limitations, including dataset bias toward well-characterized viruses and the lack of standardized benchmarking protocols, thereby hindering reproducibility and reducing the reliability of model comparisons across the different studies [34].

### 2.2. Imaging-Based Viral Detection Using Deep Learning

AI-driven analysis of biomedical images has become an essential feature in viral diagnostics, particularly in clinical contexts. In more detail, deep learning models, especially convolutional neural networks, have achieved high accuracy in detecting infection patterns in radiological images such as chest X-rays and CT scans, having been widely applied during the COVID-19 pandemic for rapid screening and severity assessment [35,36].

Beyond radiological imaging, AI has been successfully applied to histopathology and microscopy for detecting virus-induced cellular alterations. Interestingly, transfer learning approaches using pre-trained architectures such as ResNet and EfficientNet have proven particularly effective in addressing limited dataset sizes while maintaining high diagnostic performance [37,38].

In contemporary studies, transformer-based and hybrid deep learning models have been introduced to improve global feature extraction in medical images. These architectures offer enhanced contextual understanding compared to traditional Convolutional Neural Networks (CNNs), although they often require larger datasets and higher computational resources [39].

Applications extend beyond COVID-19 to include Hepatitis C, where AI has been applied to liver histopathology for diagnostic support [40,41] and HIV, where neuroimaging biomarkers and radiomics approaches support diagnosis and monitoring of HIV-associated neurocognitive disorders [42,43]; AI-assisted chest imaging has also been applied to pediatric pneumonia, where automated image analysis and quality assessment improve diagnostic support in children [44,45,46].

However, imaging-based AI models face inherent limitations related to specificity. Many approaches can detect infection-related abnormalities but struggle to differentiate between viral pathogens with similar imaging signatures, clearly revealing the need for multimodal approaches that integrate imaging with genomic and clinical data [36,47].

### 2.3. Benchmark Datasets and Evaluation Metrics

The performance of AI models in viral detection is highly dependent on the availability of high-quality benchmark datasets. In genomics, repositories such as GenBank and RefSeq provide extensive viral sequence data, enabling large-scale model training. However, these datasets often exhibit class imbalance and annotation inconsistencies, which can negatively impact model performance [20].

To address emerging pathogens, platforms such as GISAID have become central to global genomic surveillance, particularly during the COVID-19 pandemic, by providing rapid access to viral sequences for AI-driven analysis [48]. Similarly, Nextstrain integrates genomic and epidemiological data to visualize viral evolution in real time, supporting both outbreak monitoring and comparative benchmarking [49].

In the imaging domain, datasets such as COVIDx and other curated repositories have supported the rapid development of AI-based diagnostic tools. Despite their usefulness, many of these datasets lack diversity and are often collected under specific clinical conditions, raising concerns about generalizability [50].

Evaluation metrics such as accuracy, precision, recall, F1-score, and AUC-ROC are widely used to assess model performance. However, these metrics may not fully capture model robustness, particularly in real-world and imbalanced scenarios. Consequently, recent studies emphasize the importance of external validation, cross-dataset testing, and uncertainty estimation [31].

A major limitation in this field is the lack of standardized benchmarking protocols. Variations in preprocessing, dataset splitting, and evaluation strategies make it difficult to compare results across studies. Initiatives promoting FAIR data principles (Findable, Accessible, Interoperable, Reusable) aim to improve reproducibility and transparency in AI-based virology research [51].

### 2.4. Key Challenges and Research Gaps

Despite remarkable advances in AI-based viral detection, several methodological challenges continue to limit model robustness, generalizability, and clinical translation. Data scarcity and imbalance remain critical issues, particularly for emerging viral pathogens, leading to reduced model generalization and increased risk of overfitting [20,34].

Model interpretability is another major concern, with many deep learning models operating as black boxes, which clearly limits their acceptance in clinical settings where explainability is an essential prerequisite. Thus, recent efforts to incorporate explainable AI (XAI) techniques, such as SHAP (SHapley Additive exPlanations) and LIME (Local Interpretable Model-agnostic Explanations), have shown promise in improving transparency and clinical trust [47,52]. Recent advances in XAI have introduced several complementary techniques for improving model transparency. SHAP quantifies the contribution of individual input features to model predictions, making it particularly useful for genomic analyses and biomarker identification. LIME provides local approximations of complex models, allowing clinicians to understand the rationale behind individual predictions irrespective of the underlying algorithm. For image-based virology applications, Gradient-weighted Class Activation Mapping (Grad-CAM) [53,54] generates visual heatmaps that highlight image regions contributing most strongly to diagnostic decisions, facilitating verification that predictions are based on biologically relevant structures rather than imaging artefacts or spurious correlations. Collectively, these explainability methods enhance transparency, support error analysis, increase clinician confidence, and may facilitate regulatory acceptance of AI-assisted diagnostic systems in virology.

Data heterogeneity and lack of integration also pose significant challenges, as most current approaches analyze genomic, imaging, and clinical data separately, despite their complementary nature. In this context, emerging multimodal AI frameworks aim to address this issue by integrating heterogeneous data sources, demonstrating improved performance and robustness [55,56].

Equally important, reproducibility remains a critical issue due to inconsistencies in datasets, methodologies, and evaluation practices, and addressing these challenges requires the adoption of open datasets, standardized protocols, and transparent reporting standards. In this frame, initiatives such as reproducibility checklists and FAIR data principles are increasingly emphasized in biomedical AI research [51,57,58]. The principal AI approaches used for viral pathogen detection and classification, together with their strengths, limitations, and representative applications, are summarized in Table 1.

Overall, genomic and imaging approaches each provide distinct strengths to viral detection, with genomics offering sequence-level specificity, while imaging provides rapid, non-invasive diagnostics. Yet both approaches share common obstacles, particularly around data quality and reproducibility, which remain unresolved in many studies [34,61].

Among the AI methodologies reviewed, no single approach consistently outperforms the others across all virological applications. Conventional machine-learning algorithms, such as support vector machines and random forests, remain advantageous when datasets are relatively small, structured, and interpretable, making them suitable for biomarker discovery and clinical decision support. In contrast, deep learning approaches, particularly convolutional neural networks and transformer-based architectures, generally achieve superior predictive performance when large annotated datasets are available, especially for genomic sequence analysis and medical imaging [62]. However, these models require substantially greater computational resources and often exhibit reduced interpretability. Multimodal AI frameworks (Table 1) represent a promising compromise by integrating genomic, imaging, and clinical information [63], thereby improving robustness and diagnostic accuracy compared with single-modality approaches. Nevertheless, their implementation is considerably more complex because of challenges related to data harmonization, interoperability, and standardized benchmarking. 

As a result, the selection of an AI methodology should depend on the specific clinical or research objective, the availability and quality of data, computational resources, and the level of model transparency required for downstream decision-making rather than on predictive performance alone. Despite encouraging results reported in many studies, relatively few AI models have undergone rigorous external validation using independent multicenter datasets. Thus, performance estimates obtained from internally validated datasets may not accurately reflect real-world clinical settings characterized by heterogeneous patient populations, imaging protocols, sequencing platforms, and laboratory procedures. Future studies should therefore prioritize prospective multicenter validation, calibration across diverse populations, and standardized benchmarking to ensure robust clinical generalizability.

After reviewing detection methods and their limitations, it becomes clear that AI’s role in virology extends further. The next section shifts focus from identifying pathogens to AI-driven approaches in viral molecular biology, protein structure prediction, host–virus interactions, and antiviral drug discovery.

### 2.5. AI for Viral Molecular Biology, Protein Structure Prediction, Host–Virus Interactions, and Antiviral Drug Discovery

**Figure 2:**
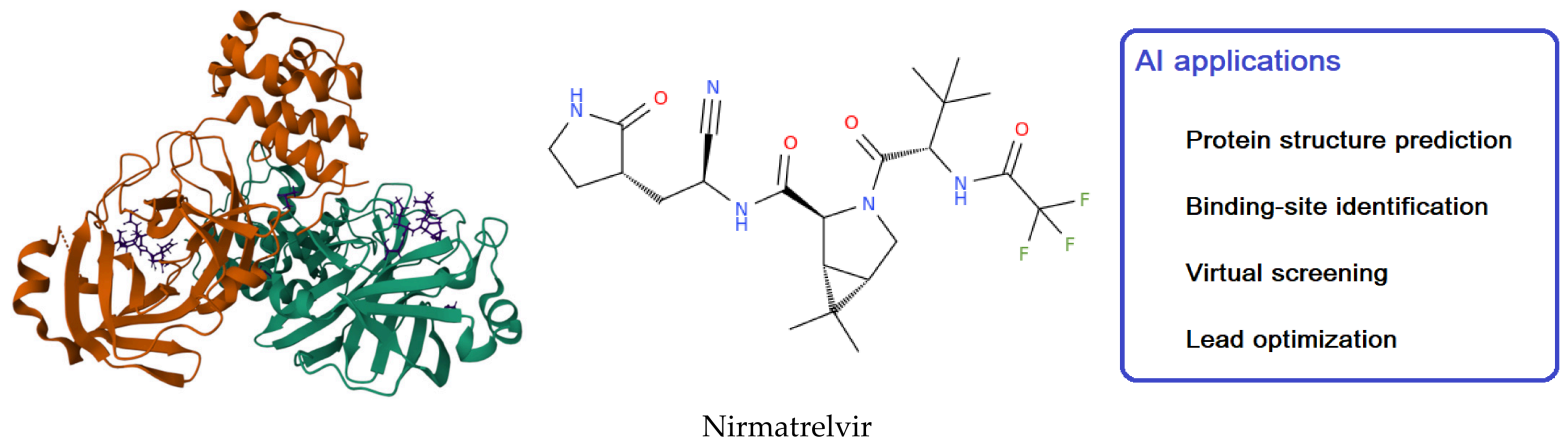
Crystal Structure of SARS-CoV-2 MPro in complex with nirmatrelvir [71] (PDB ID: 8E25; structure freely available at the link https://www.rcsb.org/structure/8E25, accessed on 11 June 2026), and AI applications in protein-targeting antiviral strategies.

Current progress in artificial intelligence has expanded the role of computational methods beyond pathogen detection and surveillance, enabling new approaches for understanding viral molecular biology and accelerating antiviral drug discovery. Machine learning and deep learning models are increasingly used to predict protein structure, characterize host–virus interactions, identify therapeutically relevant biomolecular targets, and support the design of new antiviral compounds [64,65]. These developments have become particularly important in the context of emerging viral pathogens, where rapid characterization of viral proteins and host responses was essential for guiding experimental research and therapeutic development. The SARS-CoV-2 main protease (Mpro) [66,67,68,69,70] is shown as an example of a therapeutically relevant viral target (Figure 2). Predicted structural information can subsequently guide virtual screening, molecular docking, and lead optimization, accelerating the discovery and evaluation of antiviral compounds. This integrated workflow illustrates how AI moves beyond pathogen detection and surveillance to enable deeper molecular understanding and therapeutic innovation (Figure 2).

#### 2.5.1. AI for Viral Protein Structure Prediction

Protein structure is a fundamental determinant of viral function, influencing processes such as host–cell entry, replication, immune evasion, and drug susceptibility. Traditionally, pathogenic protein structures have been determined using experimental techniques including X-ray crystallography, nuclear magnetic resonance spectroscopy, and cryo-electron microscopy [72]. Although highly accurate, these approaches are often time-consuming and resource-intensive.

In this context, the emergence of deep learning-based structure prediction systems has transformed structural virology. Models such as AlphaFold [16] and related protein language models can predict three-dimensional protein conformations with unprecedented accuracy, enabling rapid characterization of viral proteins even in the absence of experimentally determined structures. These approaches have been widely applied to proteins from SARS-CoV-2, influenza viruses, HIV, hepatitis viruses, and numerous emerging zoonotic pathogens [14], with structural predictions providing valuable insights into receptor binding, viral assembly, antigenic variation, and potential drug-binding pockets. In addition to individual protein structures, AI methods increasingly support the prediction of protein complexes [15], facilitating the study of viral replication machinery and interactions between viral and host proteins [13]. Such information is critical for identifying vulnerable molecular targets suitable for therapeutic intervention.

#### 2.5.2. Prediction of Host–Virus Interactions

Successful viral infection depends on a complex network of interactions between viral proteins and host cellular factors [73]. Thus, understanding these biomolecular interactions is essential for elucidating mechanisms of pathogenesis and identifying host-directed therapeutic targets.

In this setting, machine learning approaches have been developed to predict protein–protein interactions using sequence features, structural information, evolutionary conservation, and biological network data [74]. Deep neural networks and graph-based learning models can integrate heterogeneous datasets to identify previously unknown host factors involved in various processes including viral entry, replication, and immune modulation [75].

Applications have been reported for numerous viral pathogens, including HIV, influenza viruses, hepatitis viruses, Ebola virus, and SARS-CoV-2 [76,77,78].

During the COVID-19 pandemic, AI-assisted analyses contributed to the identification of host proteins associated with viral replication pathways and inflammatory responses [79], and these findings provided valuable information for drug repurposing and the development of host-targeted antiviral strategies [80].

Network-based approaches further enable the construction of host–virus interactomes, revealing critical molecular hubs that may represent attractive therapeutic targets [81]. These systems-level analyses provide a broader understanding of viral pathogenesis than conventional single-gene studies.

#### 2.5.3. AI-Assisted Antiviral Drug Discovery

Drug discovery based on both exploration of synthetic derivatives and natural compounds [67,82,83,84,85,86] is traditionally a lengthy and expensive process characterized by high attrition rates [87]. Artificial intelligence is increasingly used to accelerate multiple stages of antiviral development, including target identification, virtual screening, lead optimization, and drug repurposing [88]. Notably, machine learning models can rapidly screen large chemical libraries to identify compounds with predicted antiviral activity [89], with structure-based approaches combining protein structure prediction with molecular docking and binding-affinity estimation, enabling the prioritization of candidate molecules before experimental validation [90]. Deep generative models further support the design of novel chemical entities endowed with optimized pharmacological properties.

Several viral targets have been investigated using AI-assisted approaches, including the above-mentioned SARS-CoV-2 Mpro, but also other key viral enzymes such as RNA-dependent RNA polymerase, HIV protease, influenza neuraminidase, and hepatitis virus enzymes [91,92,93,94]. During the COVID-19 pandemic, AI-driven virtual screening campaigns identified multiple compounds for subsequent experimental evaluation and contributed to the development of antiviral therapeutic strategies [95].

Beyond de novo drug discovery, AI has proven valuable for drug repurposing [96]. This strategy has already demonstrated its utility against emerging viral pathogens. For example, during the 2022 monkeypox outbreak caused by Monkeypox virus, AI-driven drug repurposing frameworks integrating deep learning with structure-based computational approaches successfully identified FDA-approved and investigational compounds targeting viral proteins, highlighting the potential of AI to rapidly screen therapeutic candidates during public health emergencies [96]. In other words, by integrating molecular, clinical, and pharmacological datasets, computational models can identify approved drugs with potential antiviral activity, reducing development costs and shortening the time required for clinical deployment.

Together, the current advances in protein structure prediction, host–virus interaction modelling, and antiviral drug discovery complement the several AI-based detection and classification approaches by extending the analytical pipeline from initial pathogen identification to molecular characterization and therapeutic targeting. This continuity highlights how AI methods operate across the full virological spectrum, from recognizing viral signatures in genomic and imaging data to informing downstream structural analysis, drug development, and ultimately outbreak forecasting.

## 3. Automated Quality Assessment of Virology-Related Digital Content

**Figure 3:**
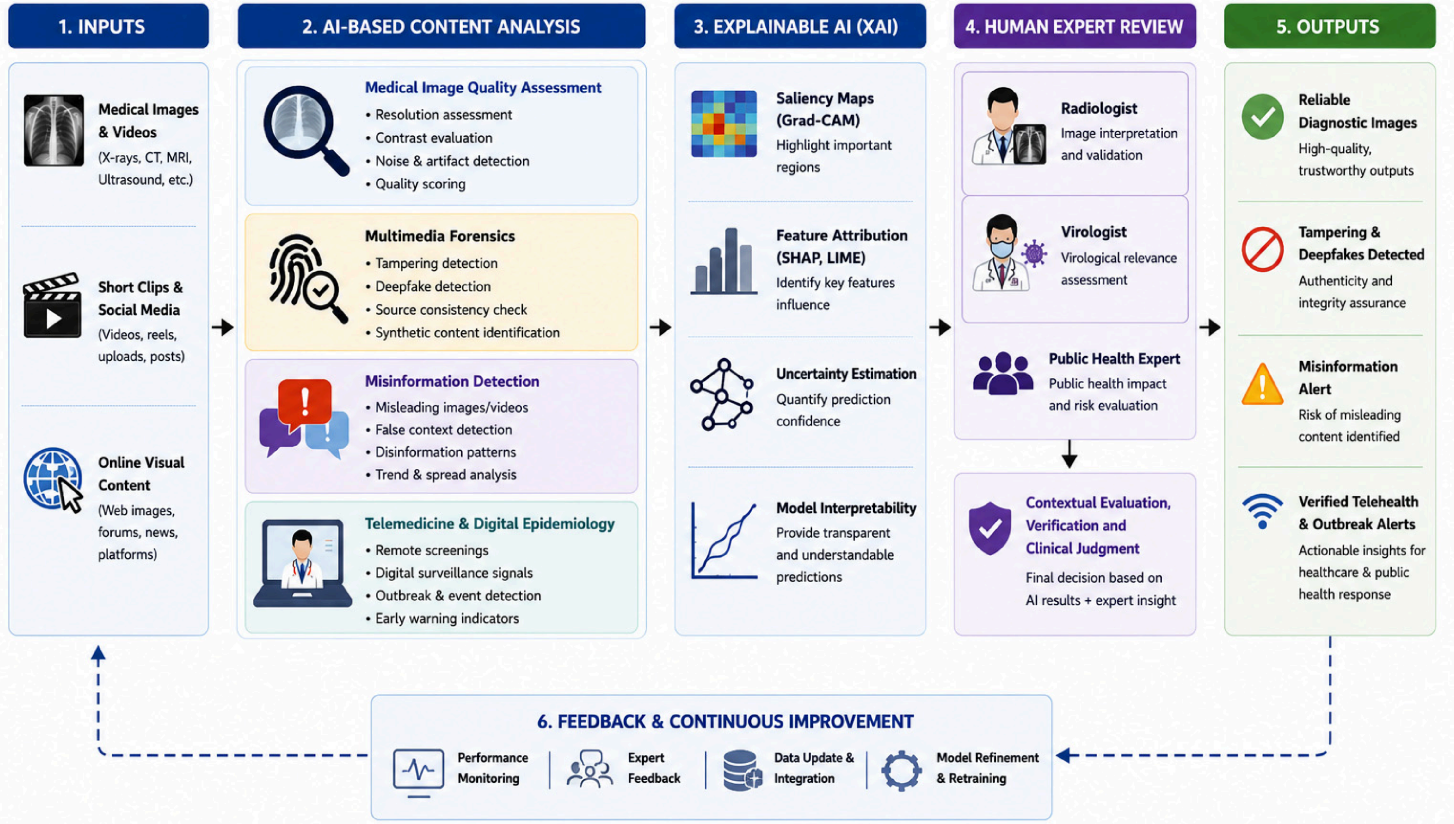
Workflow illustrating an AI-based quality assessment and trust framework in virology, generating reliable diagnostic images, detecting tampering and deepfakes, issuing misinformation alerts, and verifying telehealth inputs and outbreak notifications (image created by the authors using the Canva online platform, https://www.canva.com/, accessed on 11 July 2026).

Automated medical image quality assessment (MIQA) is particularly important during viral outbreaks [97], where rapid acquisition of large volumes of heterogeneous imaging data across multiple healthcare settings can compromise diagnostic consistency and downstream AI performance. In fact, while AI has transformed viral detection, molecular characterization, and outbreak surveillance, its effectiveness ultimately depends on the quality, authenticity, and reliability of the digital information on which these systems are trained and deployed. Consequently, growing attention has been directed toward AI-driven approaches for assessing medical image quality, detecting manipulated content, identifying misinformation, and supporting trustworthy digital health ecosystems. This section examines AI techniques for assessing the reliability, authenticity, and diagnostic quality of virology-related digital content, including user-generated medical images, short videos, and online visual media. Indeed, with the rapid proliferation of digital health information, ensuring the integrity and quality of such content has become critical in both clinical and public health contexts. In this context, contemporary advancements in deep learning, multimedia forensics, and multimodal analysis enable automated detection of artefacts, synthetic media, and misleading visual representations of infectious diseases, particularly highlighted during the COVID-19 pandemic (see Figure 3) [98,99].

### 3.1. AI-Based Medical Image Quality Assessment

As discussed earlier, automated MIQA is an essential tool for reliable AI-assisted diagnosis. Deep learning methods, especially convolutional neural networks, show strong performance across chest X-ray and computed tomography (CT) imaging, both widely used in viral infection diagnosis [100,101]. These systems provide objective, reproducible quality evaluation, reducing the subjectivity of manual inspection. Recent work emphasizes no-reference image quality assessment (NR-IQA), which operates without ground-truth images and is better suited for clinical deployment. Deep neural network models can learn distortion patterns from noise, motion artefacts, or compression, offering greater robustness than traditional handcrafted methods [102,103]. 

Comparative analyses of image enhancement methods applied to COVID-19 CT scans further illustrate these challenges. Although objective image-quality metrics provide useful benchmarks, they do not always correlate with radiologists’ assessments of diagnostic quality [97]. In particular, enhancement algorithms that improve global image contrast may inadvertently suppress clinically relevant features, revealing the need for medical image quality metrics that prioritize diagnostic information rather than generic visual quality. These findings highlight the importance of developing AI-driven quality assessment frameworks specifically tailored to medical imaging applications [97]. Applications of MIQA span a wide range of viral infections. Radiology pipelines for influenza-associated pneumonia rely on high-quality chest imaging to ensure reliable AI-assisted diagnosis and clinical interpretation [104]. In pediatric contexts, automated image quality assessment has been applied to chest radiography for pediatric pneumonia, where high-quality images improve the reliability of AI-assisted diagnosis and clinical interpretation [105,106]. 

In tropical medicine, ultrasound imaging for Dengue virus requires quality control to ensure accurate detection of plasma leakage and organ involvement [107]. High-quality neuroimaging is essential for the accurate assessment of congenital brain abnormalities associated with Zika virus infection, supporting reliable AI-assisted image interpretation [106]. In chronic viral diseases, reliable MRI acquisition is essential for AI-based neuroimaging analysis, enabling the extraction of imaging biomarkers for the diagnosis and monitoring of HIV-associated neurocognitive disorders [42,43,108], while histopathology image scoring enhances diagnostic reliability in HCV liver biopsies [40,41].

However, several limitations persist. Dataset variability, annotation inconsistencies, and domain shifts between imaging devices can significantly affect model generalization. Moreover, deep models are prone to shortcut learning, where predictions rely on spurious correlations rather than clinically meaningful features, raising concerns about reliability in high-stakes medical environments [109,110]. Additionally, conventional image-quality metrics may fail to reflect clinically meaningful image quality, emphasizing the need for evaluation frameworks that incorporate expert interpretation and diagnostic relevance.

### 3.2. Multimedia Forensics, Synthetic Media Detection and Trustworthy Medical AI

The growing availability of manipulated and AI-generated medical content makes multimedia forensics essential in virology and digital health. Generative adversarial networks (GANs) can now create highly realistic synthetic medical images, including CT scans, chest X-rays, and histopathology slides, that may misrepresent disease conditions and compromise diagnostic reliability [111,112]. To counter these risks, deep learning-based forensic approaches have been developed, relying on convolutional neural networks and transformer architectures to identify subtle artefacts introduced during image manipulation. 

Large benchmark datasets such as Face Forensics have facilitated the training of robust detection models [113,114], while newer medical-specific datasets have expanded coverage to multiple imaging modalities, improving detection accuracy in clinical contexts [115]. Nevertheless, the field remains characterized by an arms race: as GANs and diffusion models evolve to produce increasingly convincing synthetic medical images, forensic detection systems must continuously adapt to remain effective [116].

Applications in medical imaging highlight the urgency of these methods, as synthetic chest X-rays, CT scans, histopathology slides, and other AI-generated medical images may misrepresent disease conditions and compromise diagnostic reliability [115,116,117]. Such manipulated content also poses risks for public communication, where misleading visual evidence can contribute to health misinformation and reduce trust in clinical decision-making [111,118]. Deep learning models may inadvertently exploit acquisition-specific artifacts, scanner-dependent noise patterns, or other non-biological features that correlate with disease labels, rather than learning clinically meaningful characteristics. 

This challenge, highlighted in studies of COVID-19 chest imaging [119], revealed the need for explainable AI, rigorous dataset curation, and forensic validation to ensure that diagnostic models rely on biologically relevant information rather than spurious correlations. Despite promising progress, challenges persist, including limited generalization to unseen manipulations, dataset bias, and the need for multimodal forensic pipelines that combine image analysis with metadata and contextual signals [118]. Addressing these issues will be essential to safeguard both clinical decision-making and public trust in digital health information.

### 3.3. AI for Misinformation Detection in Visual Health Content

Beyond technical image quality, AI plays a crucial role in assessing the credibility of virology-related content circulating online. The COVID-19 pandemic highlighted the impact of misinformation, often referred to as an “infodemic”, on public health decision-making and risk perception [120]. Recent approaches integrate computer vision with natural language processing to build multimodal AI systems capable of analyzing both visual content and contextual metadata. These systems can detect misleading or falsely labeled medical images more effectively than unimodal models [115,118].

For instance, multimodal misinformation detection frameworks combining visual content, textual information, and metadata have demonstrated improved robustness compared to text-only or image-only approaches [118,121,122]. Despite promising progress, misinformation detection remains challenging. Model performance depends heavily on dataset diversity and annotation quality, while limited interpretability restricts trust and adoption in clinical and public health settings. Additionally, misinformation evolves rapidly across digital platforms, requiring continuous model adaptation and adversarial robustness [117,121,123]. Emerging research also emphasizes the importance of explainable AI and cross-platform surveillance. By integrating epidemiological signals, social media metadata, and medical image verification, multimodal systems can provide early warnings of emerging misinformation trends [120,124,125]. These developments suggest the need for adaptive, transparent, and clinically reliable AI pipelines to safeguard public trust in virology-related digital content.

### 3.4. Applications in Telemedicine and Digital Epidemiology

Digital epidemiology has become an increasingly important component of modern public health by utilizing large-scale digital data generated through online platforms, search engines, and electronic health records, as well as social media to monitor disease activity and population health trends. Unlike conventional epidemiological surveillance, digital epidemiology enables near real-time detection of infectious disease outbreaks, facilitates early risk assessment, and supports rapid public health decision-making. More specifically, the widespread availability of digital information has enhanced outbreak forecasting, although challenges related to data quality, privacy protection, and interoperability remain significant considerations. During recent infectious disease emergencies, including COVID-19, digital surveillance approaches demonstrated their value by identifying changes in public information-seeking behavior and symptom-related online searches before official case reports became available. Platforms such as Google Trends and the Baidu Attention Index have been extensively investigated for monitoring diseases including influenza, COVID-19, dengue [126], Ebola, and Chikungunya [127], illustrating the growing role of AI-assisted digital surveillance in strengthening pandemic preparedness and response [128]. AI-based quality assessment systems have significant implications for telemedicine and digital epidemiology. In remote healthcare environments, ensuring the quality of transmitted medical images is essential for accurate diagnosis and treatment planning. Automated filtering of low-quality or corrupted images improves the reliability of AI-assisted diagnostic pipelines [100,103]. 

During the COVID-19 pandemic, AI-driven imaging tools were widely deployed for rapid diagnosis, emphasizing the importance of robust quality control mechanisms [129]. Furthermore, AI-based verification systems can monitor online visual content to identify misinformation trends, supporting early detection of public health risks and improving communication strategies [118]. 

Recent contributions suggest that telemedicine platforms integrating automated quality control mechanisms may improve diagnostic reliability in chest radiology and dermatology consultations, particularly in resource-limited settings [130]. Similarly, digital epidemiology frameworks that combine multimodal AI with social media surveillance have been explored to track health misinformation during infectious disease outbreaks [131,132]. Advances in federated learning further enable privacy-preserving quality assessment across distributed telemedicine networks, ensuring that sensitive patient data remains secure while maintaining diagnostic reliability [133]. At the same time, explainable AI approaches are increasingly emphasized to enhance clinician trust in automated quality scoring and misinformation detection pipelines [134]. Future directions highlight the need for adaptive telemedicine infrastructures that integrate image quality control, misinformation surveillance, and epidemiological modeling into unified AI-driven systems, thereby strengthening resilience against both diagnostic errors and digital health misinformation.

### 3.5. Key Challenges and Future Directions

Despite rapid progress, AI-based quality assessment systems face key challenges. The absence of standardized datasets and evaluation protocols complicates reproducibility and fair comparison across studies [109]. A critical challenge is detecting increasingly sophisticated synthetic media from advanced deep learning models. Current forensic techniques often fail to generalize across unseen manipulations, suggesting the need for more adaptive and robust detection frameworks [112]. Trust and interpretability are prerequisites in medical AI. Without them, even technically strong models risk rejection in clinical practice. Recent surveys highlight explainable AI methods, such as saliency mapping and concept attribution, as essential to bridge technical innovation with clinical usability [134,135]. 

Dataset bias and domain shifts remain persistent obstacles, as models trained on limited or homogeneous datasets often fail to generalize across diverse imaging environments [110]. Privacy concerns also complicate data sharing, revealing the importance of federated learning and other privacy-preserving collaborative learning strategies for model development without compromising patient confidentiality [133]. In addition, adversarial robustness is increasingly critical, as medical AI systems are vulnerable to subtle perturbations that can degrade performance or mislead clinicians [136]. Future work must therefore balance innovation with transparency, integrating explainability, fairness, and security into AI pipelines to build systems that clinicians and public health experts can reliably adopt [137,138,139]. 

A comparative overview of AI-based methods for medical image quality assessment, multimedia forensics, misinformation detection, and telemedicine applications is presented in Table 2.

Together, these approaches highlight the breadth of AI applications for digital content quality assessment in virology. MIQA ensures reliable diagnostic inputs, multimedia forensics counters manipulation risks, misinformation detection safeguards public trust, and telemedicine extends these benefits to remote care. Despite their distinct applications, all domains share common challenges, including dataset variability, explainability, privacy, and evolving adversarial threats, highlighting the need for unified AI frameworks that combine technical robustness with clinical and public health priorities.

## 4. Predictive Modelling and Surveillance for Viral Outbreak Monitoring

AI-driven predictive models have become central to outbreak detection, early warning, and epidemiological forecasting in different fields including virology. Machine learning and deep learning approaches increasingly integrate heterogeneous data sources, including clinical records, environmental signals, human mobility patterns, and social media indicators [142]. These models have demonstrated strong performance in forecasting infectious disease dynamics, particularly during the COVID-19 pandemic, where ensemble and probabilistic approaches significantly improved predictive accuracy [143,144]. Applications extend to influenza, arboviruses, and emerging respiratory diseases, highlighting the growing role of AI in public health surveillance systems [142]. Emerging innovations in graph neural networks and spatiotemporal deep learning have enabled fine-grained modelling of transmission pathways, improving outbreak localization and risk stratification. Moreover, hybrid approaches that combine mechanistic epidemiological models with machine learning have further enhanced interpretability and robustness, allowing integration of domain knowledge with data-driven insights. Furthermore, global surveillance platforms utilizing multimodal data streams have demonstrated utility for outbreak monitoring and early warning systems [145]. Future directions emphasize the importance of adaptive modelling frameworks capable of handling temporal drift, data sparsity, and adversarial misinformation signals, ensuring that predictive systems remain reliable in rapidly evolving epidemic contexts [146].

### 4.1. Machine Learning and Deep Learning for Epidemic Forecasting

Traditional compartmental models (SIR/SEIR) have evolved through machine learning techniques that capture nonlinear temporal patterns. Deep learning architectures such as long short-term memory (LSTM) networks and their variants now excel at short-term epidemic forecasting by learning temporal dependencies in infection data [147]. A growing body of work explores hybrid and embedding-based models like Epideep, which combine neural networks with epidemiological representations to improve robustness across outbreak scenarios [147,148]. Ensemble forecasting, aggregating multiple models, continues to enhance predictive reliability and reduce uncertainty in large-scale public health applications [143,149]. Recent investigations have demonstrated that attention-based deep learning models can outperform traditional recurrent networks by capturing long-range dependencies in epidemic time series. Graph neural networks have also been applied to epidemic forecasting, modeling transmission pathways across heterogeneous populations and mobility networks. Furthermore, probabilistic ensemble approaches that integrate mechanistic SEIR models with machine learning pipelines have shown improved calibration and interpretability, particularly in COVID-19 and influenza forecasting [146]. Collectively, these advances highlight the growing role of machine learning and deep learning in building adaptive, accurate, and interpretable epidemic forecasting systems.

### 4.2. Spatiotemporal and Mobility-Driven Models

Integrating spatial and mobility data has transformed epidemic forecasting, with human movement patterns derived from mobile phone records, transportation networks, and global mobility datasets revealing transmission dynamics that traditional models often miss [150,151]. Mobility-informed frameworks now are able to capture regional and global spread with remarkable precision, supporting early detection and targeted interventions [142]. Spatiotemporal graph neural networks (GNNs) model geographic interactions as dynamic graphs, enabling nuanced forecasts of disease propagation and improving outbreak localization. Recent work has demonstrated that mobility-driven machine-learning–based approaches can predict influenza [152] and COVID-19 spread by incorporating mobility patterns that improve the characterization of transmission dynamics [153]. Mobility-informed epidemiological models have enhanced understanding of disease spread and intervention effects [154]. In parallel, privacy and ethical concerns remain active areas of debate, as the use of mobile phone and transportation data raises questions about surveillance and data governance [155]. Advances in federated learning and differential privacy offer promising solutions, enabling epidemic forecasting while preserving individual confidentiality [133]. Future directions emphasize the need for transparent, adaptive spatiotemporal frameworks that balance predictive accuracy with ethical safeguards, ensuring that mobility-driven AI systems can be trusted in public health practice [156].

### 4.3. Multimodal Data Integration for Surveillance

Modern surveillance systems increasingly rely on multimodal data: epidemiological, environmental, demographic, and digital signals. Integrating search queries, social media activity, and clinical reports enhances early detection [157]. Yet, over-reliance on behavioral data can introduce bias and noise, as demonstrated by the Google Flu Trends case [158], which exemplifies the pitfalls of unvalidated large-scale models. Current research emphasizes hybrid approaches that balance mechanistic epidemiology with data-driven learning to achieve both accuracy and interpretability. Latest developments in multimodal fusion frameworks combine genomic sequencing data with clinical and mobility signals, enabling more precise detection of emerging viral variants. Environmental and climatic variables, such as temperature and rainfall, have been integrated with epidemiological datasets to improve dengue and malaria risk prediction, while social media surveillance has also been explored as a complementary source of outbreak intelligence [159]. Concurrently, challenges remain in harmonizing heterogeneous data sources, ensuring interoperability across platforms, and maintaining privacy in large-scale surveillance networks. Future directions highlight the need for standardized multimodal benchmarks and explainable integration strategies in order to ensure that surveillance systems remain both accurate and trustworthy in public health practice [160].

### 4.4. Applications in Influenza, Arboviruses, and COVID-19

AI-based forecasting has proven versatile across diverse infectious diseases. Ensemble models delivered reliable influenza predictions, utilizing multimodal datasets to capture seasonal variability and improve accuracy [149]. For arboviruses such as dengue, environmental and climatic data have been widely used for risk mapping and identification of transmission hotspots [161]. During the COVID-19 pandemic, real-time AI systems supported policy decisions by estimating transmission dynamics, case trajectories, and the impact of non-pharmaceutical interventions [144,162]. These examples illustrate the adaptability of AI-based forecasting across diverse epidemiological contexts, supporting applications ranging from respiratory to vector-borne diseases. New methodological progress in AI-based epidemic modelling has increasingly incorporated diverse data streams, including digital epidemiological signals and other multimodal surveillance data [159]. At the same time, challenges remain in harmonizing heterogeneous data sources and ensuring interpretability, highlighting the need for unified frameworks that balance technical robustness with clinical and public health priorities [160].

### 4.5. Challenges and Future Research Directions

Despite significant progress, several challenges persist in AI-based epidemic forecasting. Data sparsity, reporting delays, and inconsistencies across regions can negatively impact model performance. Moreover, temporal drift, where model accuracy degrades over time due to changing transmission dynamics, remains a critical issue in long-term forecasting [143,162].

Model interpretability is another major concern, as many deep learning models operate as black boxes, limiting their adoption in public health decision-making. Therefore, there is increasing demand for interpretable and transparent AI systems that provide actionable insights rather than purely predictive outputs [163].

Future research should focus on developing hybrid models that integrate mechanistic epidemiological knowledge with data-driven learning approaches. Standardized evaluation frameworks, open and interoperable data-sharing practices, and interdisciplinary collaboration will be essential for advancing trustworthy, reproducible, and clinically relevant AI-driven epidemic surveillance systems.

## 5. Conclusions

Artificial intelligence is increasingly integrated into virology, offering practical tools for pathogen detection, image-based diagnostics, digital-content evaluation, and outbreak surveillance. Machine-learning and deep-learning models can support established laboratory and epidemiological workflows by improving classification accuracy, reducing manual workload, and enabling the analysis of heterogeneous data sources. Beyond pathogen detection and surveillance, AI is increasingly transforming molecular virology through advances in protein structure prediction, host–virus interaction modelling, and antiviral drug discovery. The integration of protein language models, structural biology, and multimodal learning frameworks offers new opportunities to accelerate target identification and therapeutic development. As research progresses, the most promising developments appear to be those that combine AI with domain knowledge, standardized evaluation pipelines, and careful validation across diverse settings. Beyond technical performance, successful translation of AI into routine virology also requires careful consideration of regulatory, organizational, and clinical implementation aspects. Importantly, AI-based diagnostic systems intended for clinical use must comply with evolving regulatory frameworks governing medical devices and software as medical devices, while also demonstrating safety, robustness, reproducibility, and continuous post-deployment monitoring. Furthermore, integration into existing laboratory information systems, hospital workflows, and public health surveillance infrastructures remains a significant challenge that extends beyond algorithm development. Additional barriers include interoperability among heterogeneous data platforms, limited clinician familiarity with AI systems, infrastructure costs, cybersecurity concerns, and the need for continuous model updating as viral epidemiology evolves. To translate the recent developments of AI in virology into routine research and clinical practice, several actionable priorities should guide future work. First, the development of large, diverse, and standardized benchmark datasets, accompanied by harmonized annotation protocols and external validation frameworks, is essential to improve model robustness and reproducibility. Second, greater emphasis should be placed on explainable and uncertainty-aware AI methods that provide transparent predictions and facilitate adoption by clinicians, laboratory scientists, and public health authorities. Third, multidisciplinary collaborations should be strengthened to integrate expertise from virology, computational biology, artificial intelligence, epidemiology, and regulatory science, ensuring that AI systems address biologically meaningful questions and comply with ethical and governance requirements. Finally, future research should prioritize the prospective evaluation of AI models in real-world laboratory and surveillance settings, together with the development of interoperable platforms capable of integrating genomic, imaging, clinical, and environmental data.

## Figures and Tables

**Table 1:** Overview of genomic, imaging, and multimodal AI approaches for viral detection, highlighting data types, models, strengths, and limitations.

Approach Type	Data Type	Common Models	Strengths	limitations	References
Genomic AI **^†^**	DNA/RNA sequences	CNN, RNN, Transformers (e.g., DNABERT)	High specificity; ability to detect novel viruses; taxonomic classification; host–virus interaction prediction	Dataset bias toward well-characterized viruses; limited benchmarking protocols; interpretability challenges	[21,22,24,25,26]
Imaging AI	X-ray, CT, microscopy, histopathology	CNN, ResNet, EfficientNet, Transformers	Rapid, non-invasive diagnostics; strong pattern recognition; effective transfer learning for small datasets	Low pathogen specificity; variability in imaging protocols; dataset bias; high computational demand	[36,37,38,39,47]
Multimodal AI	Genomic + Imaging + Clinical data	Hybrid deep learning models	Improved robustness; integration of complementary evidence; potential for clinical deployment	Lack of standardized frameworks; heterogeneous data fusion challenges; reproducibility issues	[55,56]
Emerging Trends	Multi-source biological + digital data	Foundation models, cross-modal transformers	Potential for unified pipelines integrating genomic, imaging, and clinical streams	Need for: standardized datasets, explainable models, reproducible evaluation frameworks	[59,60]

^†^ Abbreviations: AI, Artificial Intelligence; CNN, Convolutional Neural Network; CT, Computed Tomography; DNABERT, DNA Bidirectional Encoder Representations from Transformers; EfficientNet, Efficient Neural Network architecture; ResNet, Residual Neural Network; RNN, Recurrent Neural Network; Transformer, Transformer-based neural network.

**Table 2:** Summary of AI-based methods for medical image quality assessment, multimedia forensics, misinformation detection, and telemedicine applications.

Approach Type	Application Domain	Common Models	Strengths	Limitations	References
Medical Image Quality Assessment	Chest X-rays, CT **^†^** scans, clinical imaging	CNNs, ResNet, Transformer-based IQA models	Objective, reproducible quality scoring; NR-IQA methods suitable for real-world deployment	Dataset variability; annotation inconsistencies; domain shifts across devices	[100,101,102,103,109]
Multimedia Forensics	Detection of tampered or synthetic medical images	CNNs, Transformers, forensic feature extractors	Identifies manipulation artefacts; benchmark datasets (e.g., FaceForensics++) support robust training	Arms race with GANs; limited generalization to unseen manipulations	[111,114]
Misinformation Detection	Online visual health content, social media	Multimodal AI (vision + NLP), metadata analysis	Detects misleading/falsely labeled medical images; integrates text + visuals	Rapid evolution of misinformation; dataset diversity and annotation quality critical	[118,120]
Telemedicine & Digital Epidemiology	Remote diagnostics, epidemic surveillance	CNN-based quality filters, multimodal verification systems	Improves reliability of transmitted medical images; supports early detection of misinformation trends	Lack of standardized protocols; explainability concerns in clinical deployment	[129,130,131,132]
Cross-cutting Evaluation & Validation Resources	Representative datasets across imaging, multimodal, telemedicine pipelines	Benchmarking frameworks; standardized IQA metrics; clinical validation protocols	Enables comparison across approaches; provides shared evaluation baselines; supports reproducibility and regulatory alignment	Heterogeneous dataset quality; limited clinical-grade benchmarks; sparse real-world vali dation studies	[140,141]

^†^ Abbreviations: CNN, Convolutional Neural Network; CT, Computed Tomography; GAN, Generative Adversarial Network; IQA, Image Quality Assessment; NLP, Natural Language Processing; NR-IQA, No-Reference Image Quality Assessment; ResNet, Residual Neural Network.

## Data Availability

No new data were created or analyzed in this study. Data sharing is not applicable to this article.

## Abbreviations

The following abbreviations are used in this manuscript:

AI	Artificial Intelligence
ML	Machine Learning
DL	Deep Learning
CNN	Convolutional Neural Network
RNN	Recurrent Neural Network
LSTM	Long Short-Term Memory
GNN	Graph Neural Network
NLP	Natural Language Processing
GAN	Generative Adversarial Network
IQA	Image Quality Assessment
NR-IQA	No-Reference Image Quality Assessment
SEIR	Susceptible–Exposed–Infectious–Recovered
SIR	Susceptible–Infectious–Recovered
FAIR	Findable, Accessible, Interoperable, and Reusable
GISAID	Global Initiative on Sharing All Influenza Data
CT	Computed Tomography
MRI	Magnetic Resonance Imaging
EHR	Electronic Health Record
CRISPR	Clustered Regularly Interspaced Short Palindromic Repeats
COVID-19	Coronavirus Disease 2019
SARS-CoV-2	Severe Acute Respiratory Syndrome Coronavirus 2
HIV	Human Immunodeficiency Virus
HCV	Hepatitis C Virus
HPV	Human Papillomavirus
HSV	Herpes Simplex Virus
EVD	Ebola Virus Disease
RdRp	RNA-Dependent RNA Polymerase
Mpro	Main Protease
ACE2	Angiotensin-Converting Enzyme 2
TMPRSS2	Transmembrane Protease Serine 2
PPI	Protein–Protein Interaction
XAI	Explainable Artificial Intelligence
NMR	Nuclear Magnetic Resonance
NR	No Reference
SCS	System Causability Scale
